# Clinical Anatomy for the Innervated Pattern and Boundary of the Subdeltoid Bursa

**DOI:** 10.1155/2018/4535031

**Published:** 2018-11-06

**Authors:** Chang Min Seo, Kyungyong Kim, Anna Jeon, Chang Sub Uhm, Je-Hun Lee, Seung-Ho Han

**Affiliations:** ^1^Department of Anatomy, College of Medicine, Chung-Ang University, Seoul, Republic of Korea; ^2^Department of Anatomy, College of Medicine, Korea University, Seoul, Republic of Korea; ^3^Anatomy laboratory, College of Sports Science, Korea National Sport University, Seoul, Republic of Korea

## Abstract

The aim of this study was to accurately identify the distribution of sensory nerve branches running to bursa with mesoscopic dissection and boundaries following the injection of gelatin into the bursa. Eighteen shoulders of 11 Korean soft cadavers (average age, 65 years; age range, 43 - 88 years) were dissected. The most prominent point of greater tubercle of the humerus (GT) was used as a reference point. The horizontal line passing through GT was used as the x-axis while the vertical line passing through the GT was used as the y-axis. Average distances of the anterior, posterior, superior, and inferior from the GT were 1.9±0.6, 2.4±1.3, 2.1±0.7, and 3.2±1.5 cm, respectively. In 15 cases of 18 shoulders, the anterior branch of the axillary nerve was distributed to the subdeltoid bursa that was running posteriorly. The muscular branch of the anterior and middle parts of the deltoid was distributed to the branch of nerve that was running into the subdeltoid bursa. A branch of the posterior cord of brachial plexus was distributed to the subdeltoid bursa that was running anteriorly in three cases. Most of the branches of the axillary nerve were distributed into the posterolateral area. The branches of the posterior cord of brachial plexus were distributed in the anterolateral area. These results might be useful for preventing residual pain on the anterior shoulder region following an injection for the relief of shoulder pain.

## 1. Introduction

Acute and chronic pain may occur in the shoulder region due to various factors related to individual structures. Shoulder pain can markedly decrease the quality of life of a patient. Pain caused by disorders involving the shoulder increases with age [1, 2].

Shoulder pain is usually caused by the anterior, lateral, and posterior of the shoulder region as well as muscle, ligament, bursa, and capsule which constitute the shoulder joint [3, 4]. Patients with a shoulder disorder usually complain of pain on the lateral region of the shoulder that is often caused by a bursa [3, 5].

The bursa is a structure that makes up the shoulder. It is located in joints, tendons, and bones. It is one of the main structures of the body [6]. When the shoulder moves, the bursa acts a smooth movement to the joint in order to reduce friction so that the joint can move freely, allowing shoulder internal rotation and elevation [7]. Bursitis of the shoulder region occurs when the bursa is inflamed, thus restricting movement due to shoulder pain. It may cooccur with other diseases such as impingement syndrome, rotator cuff tendinitis, and tears. Direct trauma to the bursa can occur as well [8, 9].

The main bursae of the shoulder are subacromial and subdeltoid bursa, which are the largest in the human body [10]. Most studies and anatomy literatures have suggested that subacromial and subdeltoid bursa are separated. However, a few studies have disagreed with this [11–14]. Previous studies on subacromial and subdeltoid bursa have demonstrated regional locations of the bursa using imaging techniques such as magnetic resonance imaging and computed tomography [13]. However, these studies only used medical images.

In cadaver studies, the bursa of human body has been directly investigated in order to determine its shape by injecting latex or serum [12–15]. However, anatomical information on the size and location of subacromial or subdeltoid bursa remains unclear.

Research has been conducted on sensory nerve innervation in order to study bursal pain [3, 6, 16]. The suprascapular nerve has been identified as the main nerve running to and innervating the subacromial bursa [6, 16]. Other researchers have demonstrated that the lateral pectoral nerve also continually innervates the subacromial bursa [16]. A recent study has reported that more than 60% of the axillary nerve branches innervate the subacromial bursa [3]. An alternative explanation for persistent shoulder pain is that the source of the pain is peripheral nerve origin. The peripheral nerves that innervate the bursa may have been subject to damage, either at the time of initial trauma or through subsequent surgical intervention. There have been a few published studies on subacromial bursa, but there has been little research on subdeltoid bursa. Thus, this study felt that it was necessary to study the subdeltoid bursa.

Therefore, the objective of this study was to focus on subdeltoid bursa and accurately identify the distribution of sensory nerve branches running to the bursa by performing a detailed dissection of soft cadaver and examining sizes and locations by injecting gelatin into the bursa.

## 2. Materials and Methods

In the present study, 18 specimens from 11 soft cadavers were dissected. In order to find the injection site in the bursa, the skin and fat tissue of lateral region of shoulder were removed after marking the anatomical position. After removing the skin and fat tissue, we injected blue gelatin solution into the bursa. The blue gelatin solution was prepared by dissolving 20 g of gelatin (Type A: From porcine skin, Merck, Germany) in 50 ml of water and then adding blue ink (Stamp ink, Maepyo, Korea). The solution was injected into the bursa using a 50-ml syringe with an 18-gauge needle. The blue gelatin solution was kept warm at all times using a hot plate, and to solidify the solution, we waited for 20 minutes after injection in order to ensure that the dissection has been processed.

**Figure 1:**
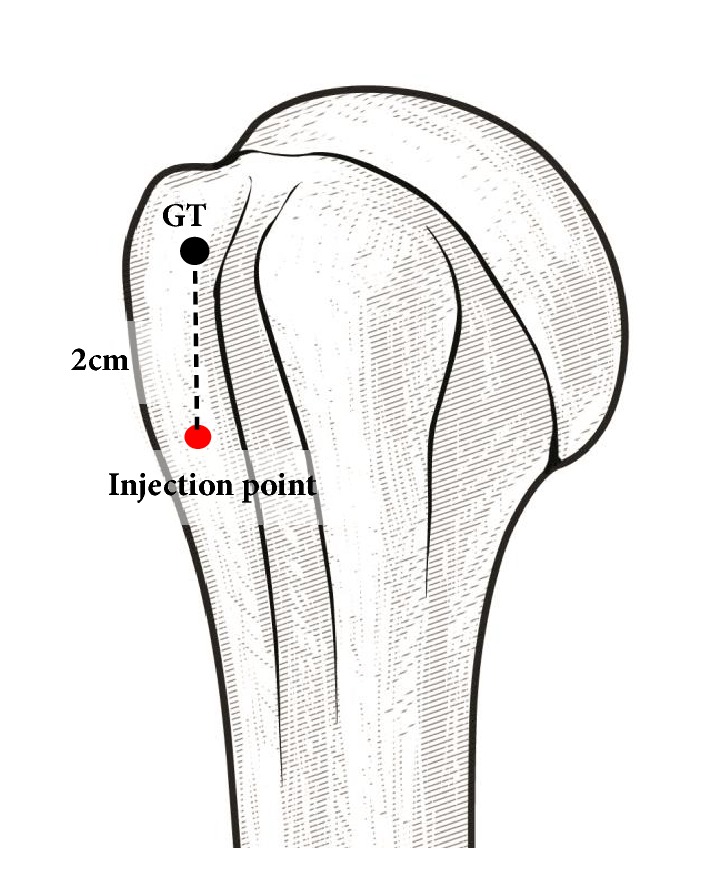
**The injection method into subdeltoid bursa.** The most prominent point of greater tubercle of the humerus (GT) was used as a reference point. Blue colored gelatin solution was injected into the subdeltoid bursa 2 cm below the GT.

From an anatomical position of looking at the front of the arm, the most prominent point of greater tubercle of humerus on the anterior view was designated as the landmark called GT. In all specimens, the gelatin solution was injected uniformly into the bursa, 2 cm below the GT (Figure 1). It did not matter which bursa was injected into, as we injected all the cadavers in the same spot. The amount of gelatin solution injected into all specimens was recorded and the mean value was calculated.

**Figure 2:**
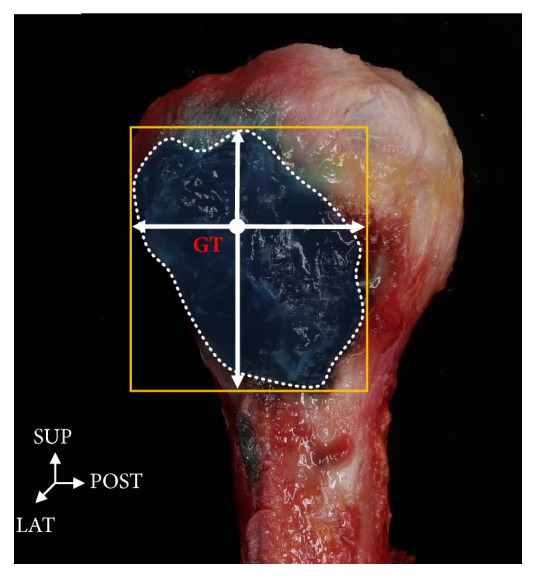
**Photograph of the lateral side of humerus.** The horizontal line passing through GT (greater tubercle) was used as the x-axis while the vertical line passing through the GT was used as the y-axis. SUP: superior, LAT: lateral, POST: posterior.

The boundaries of bursa were measured with digital calipers (Mitutoyo, Tokyo, Japan) in the anterior, posterior, superior, and inferior directions on the X-Y coordinates established relative to the GT point designated on the surface of the bursa with blue gelatin injected when the deltoid muscle was cut. The horizontal line passing through GT was used as the x-axis while the vertical line passing through the GT was used as the y-axis (Figure 2).

**Figure 3:**
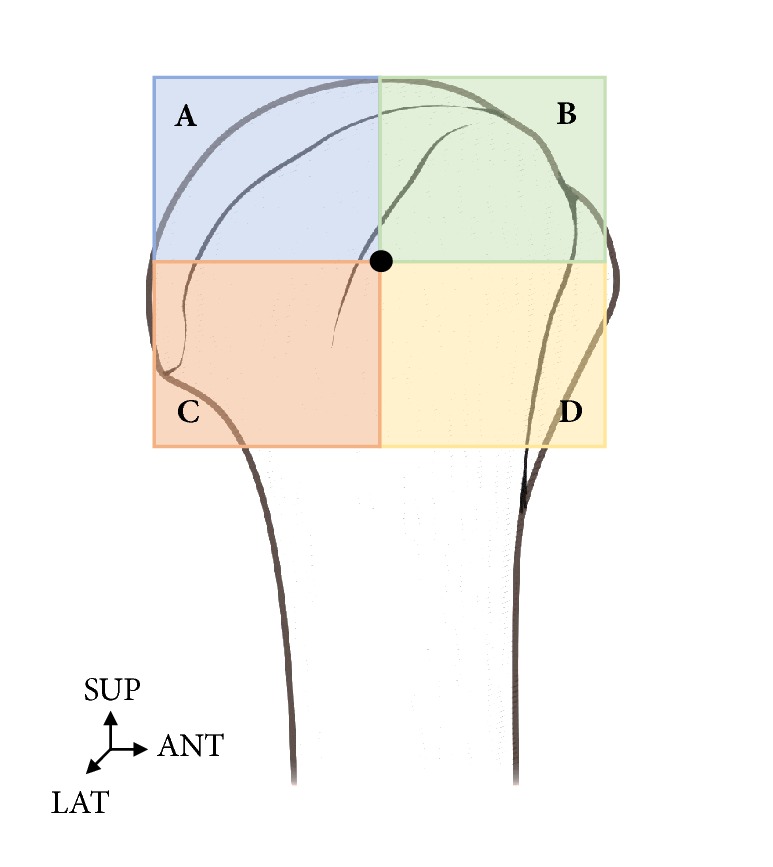
**The locational relationship of distribution of nerves that run through the bursa.** The center point when looking at the humeral head from its lateral aspect was divided into four parts (A, B, C, and D). SUP: superior, LAT: lateral, ANT: anterior.

**Figure 4:**
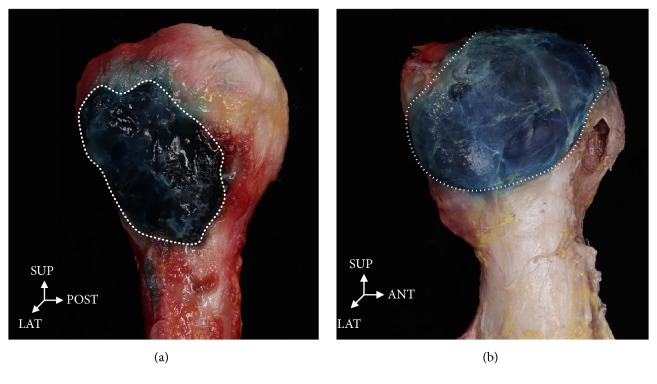
**Blue gelatin solution was injected into the bursa on the greater tubercle of humerus on lateral view.** (a) The subdeltoid bursa is divided from the subacromial bursa. (b) The subdeltoid bursa is connected to the subacromial bursa. ANT: anterior, LAT: lateral, INF: inferior, SUP: superior.

Regarding the distribution of nerves that run through the subdeltoid bursa, the center point when looking at the humeral head from its lateral aspect was divided into four parts, and nerves that branched into these parts were investigated (Figures 3 and 4).

The dissection was performed by exfoliating the skin on the neck and shoulder area. After removing the muscle fascia, the clavicular and sternal heads of the sternocleidomastoid muscle were cut close to the clavicle and sternum and then pulled upward. Subsequently, muscles in the neck were pulled back one layer at a time in order to locate the brachial plexus that extended between the scalenus anterior and medius muscles.

**Figure 5:**
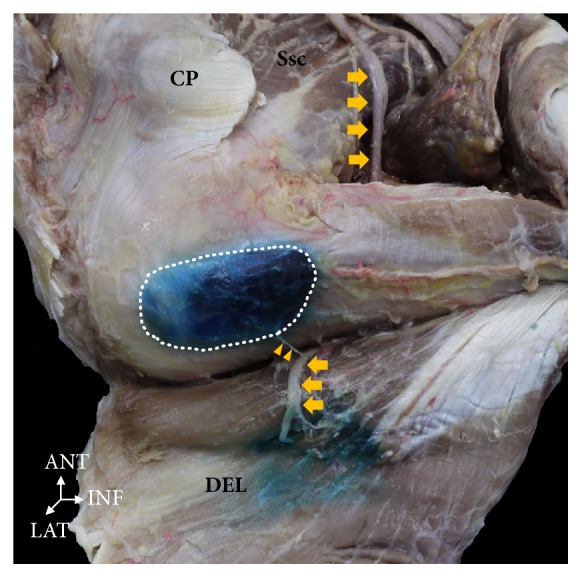
**The anterior branch of the axillary nerve (yellow arrow) distributed to the bursa running posteriorly.** The muscular branch of anterior and middle parts of the deltoid distributed to the branch of nerve that runs into the bursa (yellow triangle). CP, coracoid process; Ssc, subscapularis muscle; DEL, deltoid muscle. ANT: anterior, LAT: lateral, INF: inferior.

**Figure 6:**
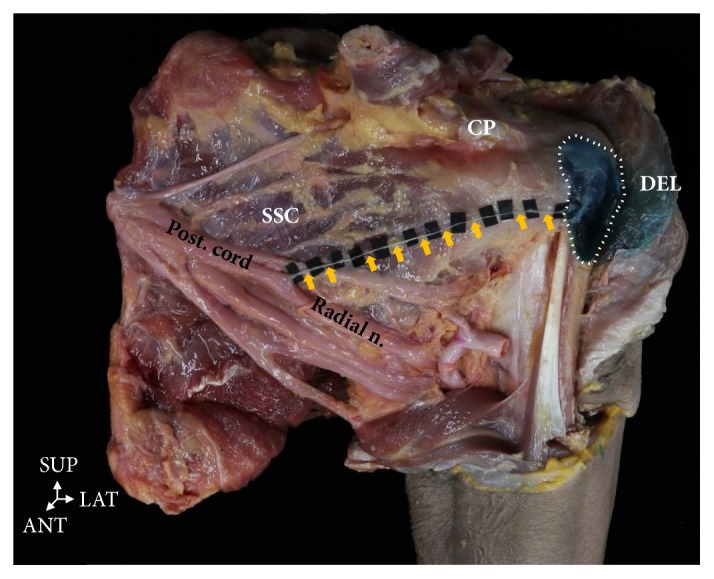
**A branch of the posterior cord of brachial plexus was distributed to the subdeltoid bursa running anteriorly (yellow arrow).** CP, coracoid process; Ssc, subscapularis muscle; DEL, deltoid muscle. ANT: anterior, LAT: lateral.

Next, connective tissues were removed, starting from those close to the body. Nerves branching from the brachial plexus were clearly identified. Nerves that branched from the axillary nerve and posterior cord of brachial plexus and ran through the subdeltoid bursa were located and dissected. Fine dissection by anatomical experts can lead to tracing of the sensory nerve to the bursa (Figures 5 and 6).

## 3. Results

### 3.1. Boundaries of the Subdeltoid Bursa

As a result of injecting gelatin into the bursa of 18 shoulders, separation of the subacromial bursa and subdeltoid bursa was observed in 16 (89%) shoulders. Meanwhile, the subacromial bursa and subdeltoid bursa were connected in two (11%) shoulders (Figure 4). Except for the two cases in which the subacromial bursa and subdeltoid bursa were difficult to distinguish as they were connected together, boundaries of the subdeltoid bursa were identified in the remaining 16 shoulders. Meanwhile, nerve distribution was properly identified in all 18 shoulders.

An examination of the boundaries of the subdeltoid bursa in 16 samples showed that distances of the anterior, posterior, superior, and inferior from the GT were 1.9±0.6, 2.4±1.3, 2.1±0.7, and 3.2±1.5 cm, respectively (Table 1). In all samples, the subdeltoid bursa basically had an oval shape. However, this shape was irregular, making it difficult to classify them into different types. The volume of gelatin injected had a mean value of 6.1 ml.

### 3.2. Distribution of Nerves

Two types of nerves branched to the subdeltoid bursa. The first type originated from the axillary nerve. The axillary nerve that branched from the brachial plexus went around and ran behind the humeral neck where it bifurcated into a branch running toward the deltoid muscle and another branch running toward the subdeltoid bursa. This was observed in 15 of 18 shoulders (Figure 5).

**Figure 7:**
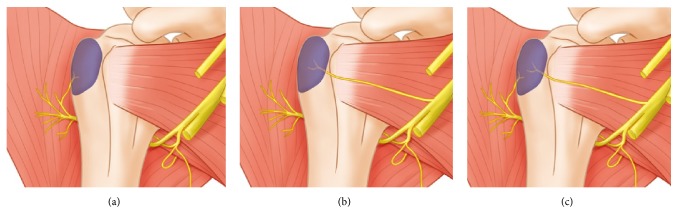
**Illustration of a branch distributed to the bursa. **Innervation of the bursa is derived from a branch of the axillary nerve (a) and a branch of the posterior cord of the brachial plexus (b). (c) is bifurcate from the axillary nerve and the posterior cord of the brachial plexus is found to enter the bursa.

The second type originated from posterior cord of brachial plexus. One branch that bifurcated directly from the posterior cord of the brachial plexus was found to run in front of the clavicle and enter the anterior of the subdeltoid bursa that had been injected with the blue gelatin solution. This was found in two of 18 shoulders (Figure 6). We also discovered cases in which branching occurred from the axillary nerve and the posterior cord of the brachial plexus. Nerves that were bifurcated from the axillary nerve and posterior cord of the brachial plexus were found to enter the subdeltoid bursa from both the anterior and posterior part. Such a case controlled by nerves on both sides was found in 1 of 18 shoulders (Figure 7).

When the distribution of nerves found in the subdeltoid bursa was expressed in percentages, 25% nerves bifurcated from the axillary nerve were distributed in the A and D zones while 50% were distributed in the C zone. Most nerves were distributed in the posterolateral area.

**Figure 8:**
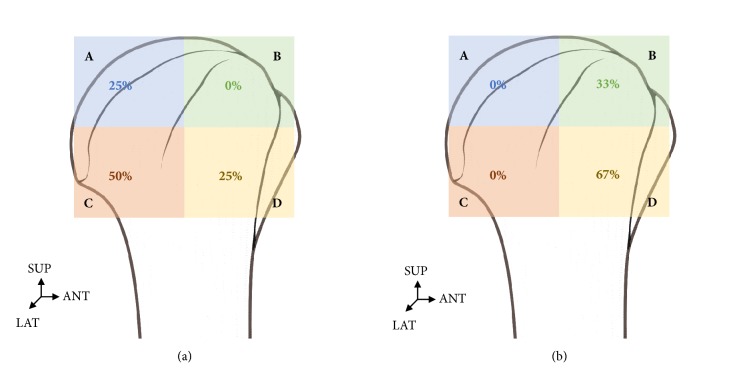
**Distribution of the axillary nerve and posterior cord of brachial plexus to the bursa.** (a) The branch of the axillary nerve was mainly in the posterolateral area. (b) The branch of the posterior cord of brachial plexus was mainly in the anterolateral area.

The distribution of nerves bifurcated from the posterior cord of the brachial plexus was 67% in C zone and 33% in B zone in three of the 18 shoulders. They were distributed mostly in the anterolateral area. In one of the 18 shoulders, nerves were distributed in both the axillary nerve and posterior cord of the brachial plexus (Figure 8).

## 4. Discussion

The subacromial and the subdeltoid bursa in the shoulder are drawn in a round shape. They are described as being connected in most atlases on human anatomy [11–15, 17]. However, the present study found that the subacromial and subdeltoid bursa were separated from each other in most cases. They existed in an oval shape, although their shape and size were irregular. One study conducted on American subjects reported that the subacromial bursa and the subdeltoid bursa are connected in 100% of cases [13], whereas another study reported that they are separated in 100% of cases [17]. However, these studies did not present pictures showing connected or separated bursae. Thus, it is difficult to accurately judge these results. In the present study, the two bursae were separated in 89% of all samples while they were connected in 11% of samples. This suggests that bursae are not connected in most people. This finding is consistent with the finding of a 1992 study by Birnbaum et al. showing that the bursae are separated in 79% of subjects but connected in 21% of subjects [12].

Previous studies have investigated nerves distributed in the bursa, focusing more heavily on the subacromial bursa rather than the subdeltoid bursa. These studies have reported that the subacromial bursa is innervated by branches of the suprascapular nerve in most cases [3, 16]. Another study has investigated nerves in the subacromial bursa and shown that the bursa is innervated by branches of the suprascapular nerve and the lateral pectoral nerve [16]. Additionally, in a recent study, Nasu et al. [3] showed for the first time that 60% of branches of the axillary nerve are distributed in the subacromial bursa. Unlike the previous studies, the present study concentrated on the subdeltoid bursa and found that the bursa was innervated by branches of the axillary nerve in 15 (83%) cases. In an additional two (11%) cases, posterior branches of the brachial plexus were distributed in the subdeltoid bursa. Such a finding has not yet been reported. The subdeltoid bursa was innervated primarily by branches of the axillary nerve and by posterior branches of the brachial plexus in some cases.

The findings of the present study provide anatomical information that may be useful for patients who experience persistent pain in their lateral shoulder, even after subacromial bursitis treatment with steroid injections and axillary block. The present study suggests that the posterior branch of the brachial plexus may innervate the subdeltoid bursa when treating pain in the lateral shoulder. Follow-up research is needed in order to further investigate anatomical injection points that block posterior branches of the brachial plexus.

## Figures and Tables

**Table 1:** Measurements of the boundaries of the subdeltoid bursa (unit: cm).

	Anterior	Posterior	Superior	Inferior
Mean ± SD	1.9±0.6	2.4±1.3	2.1±0.7	3.2±1.5

SD, standard deviation.

## Data Availability

The data used to support the findings of this study are available from the corresponding author upon request.
